# How to approach Incidentally detected Gastrointestinal Stromal Tumor during Laparoscopic Sleeve Gastrectomy: A Report of Two Cases

**DOI:** 10.5005/jp-journals-10018-1193

**Published:** 2016-12-01

**Authors:** Hakan Atas, Hakan Bulus, Gökhan Akkurt, Alper Yavuz, Utku Tantoglu, Mustafa Alimogullari, Altan Aydin

**Affiliations:** 1 Department of General Surgery, Kecioren Training and Research Hospital, Ankara, Turkey

**Keywords:** Gastrointestinal stromal tumors, Obesity, Sleeve gastrectomy.

## Abstract

Laparoscopic sleeve gastrectomy (LSG) is a frequently used bariatric surgery method. It is possible to incidentally detect gastrointestinal stromal tumors (GISTs) of stomach or other gastric tumors during this procedure. In the literature, there is limited data about cooccurrence of GIST in obesity, and treatment of such cases is still controversial. On the contrary, currently accepted treatment of local and nonmetastatic GIST cases is surgery alone, and of cases with systemic disease is surgery combined with imatinib mesylate therapy. Incidental detection of GIST during bariatric surgery may require a modification in the planned procedure, and an extensive exploration and picking a convenient surgical procedure will become necessary. Here, we present two cases – a gastric GIST and a GIST of small intestine – both of which were incidentally detected during LSG performed for obesity.

**How to cite this article:**

Atas H, Bulus H, Akkurt G, Yavuz A, Tantoglu U, Alimogullari M, Aydin A. How to approach Incidentally detected Gastrointestinal Stromal Tumor during Laparoscopic Sleeve Gastrectomy: A Report of Two Cases. Euroasian J Hepato-Gastroenterol 2016;6(2):173-175.

## INTRODUCTION

Bariatric surgery procedures are increasingly being performed as obesity becomes a global epidemic. Laparoscopic sleeve gastrectomy (LSG) is a frequently used bariatric surgery method. Gastrointestinal stromal tumor (GIST) or other tumors of stomach can incidentally be detected during this procedure. In the literature, GIST incidence is reported to be higher among obese patients who underwent bariatric surgery (0.6–0.8%), than the general population (0.0006–0.0016%).^1,2^ These incidence rates are even higher in obese patients older than 50 years old.^1,3^ For this reason, an extensive intraoperative exploration should be performed in this patient group. Complete surgical excision is still the most important treatment component in cases with incidentally detected GIST during obesity surgery. Combination with adjuvant imatinib mesylate (Gleevec, Novartis Pharma, Bengaluru, India) is the currently an accepted treatment modality for high-risk lesions or cases of systemic disease.^1,4,5^ In some cases, the localization, size, and extent of the intraoperatively detected mass may require an alteration in the intended surgical procedure. Focusing on the important aspects of the subject, we aim to present two cases with stomach and small intestine GISTs, both of which were detected incidentally during LSG performed for morbid obesity.

## CASE REPORTS

### Case 1

**Fig. 1:**
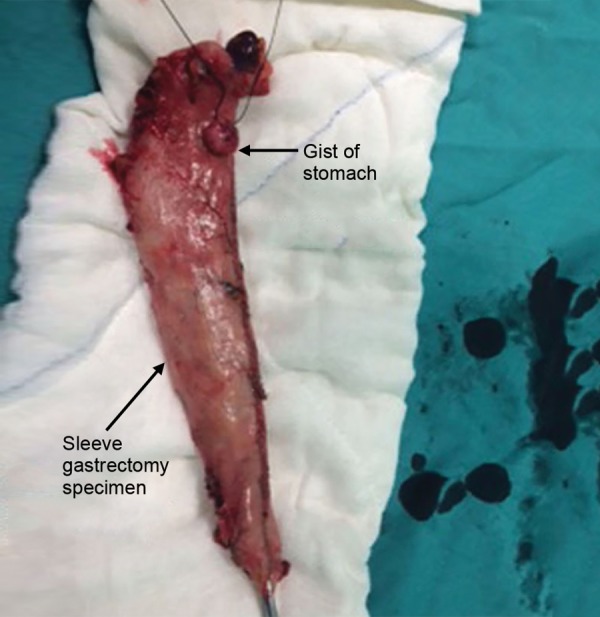
A nodular lesion located on the major curvature at the posterior side of the stomach

A 52-year-old female with a body mass index of 44 kg/m^2^ was admitted to Kecioren Training and Research Hospital, Ankara, Turkey. She did not have any accompanying disease. Routine laboratory tests, an abdominal ultrasonography (USG), and an upper gastrointestinal endoscopic examination were performed, and all of these investigations were within normal range. An LSG procedure was planned for morbid obesity. During the procedure, following the dissection of gastroepiploic vessels and omentum liberation, a nodular lesion of about 1.5 cm size with a smooth surface and off-white color located on the major curvature at the posterior side of the stomach was detected (Fig. 1). Other intra-abdominal locations were further explored and found to be clear. The initially planned gastrectomy material was already covering the lesion, and a clear surgical margin larger than 2 cm was present. Sleeve gastrectomy was completed laparoscopically. Immunohistopathologic examination demonstrated that the 1 × 1.5-cm-sized tumoral mass detected in the fundus was composed of spindle cells, showed minimal nuclear pleomorphism, did not have any mitotic activity, was positive for CD117 and CD34, and was negative for desmin and S100. A diagnosis of benign GIST was established. Expected weight loss was accomplished without any postoperative problems.

### Case 2

**Fig. 2:**
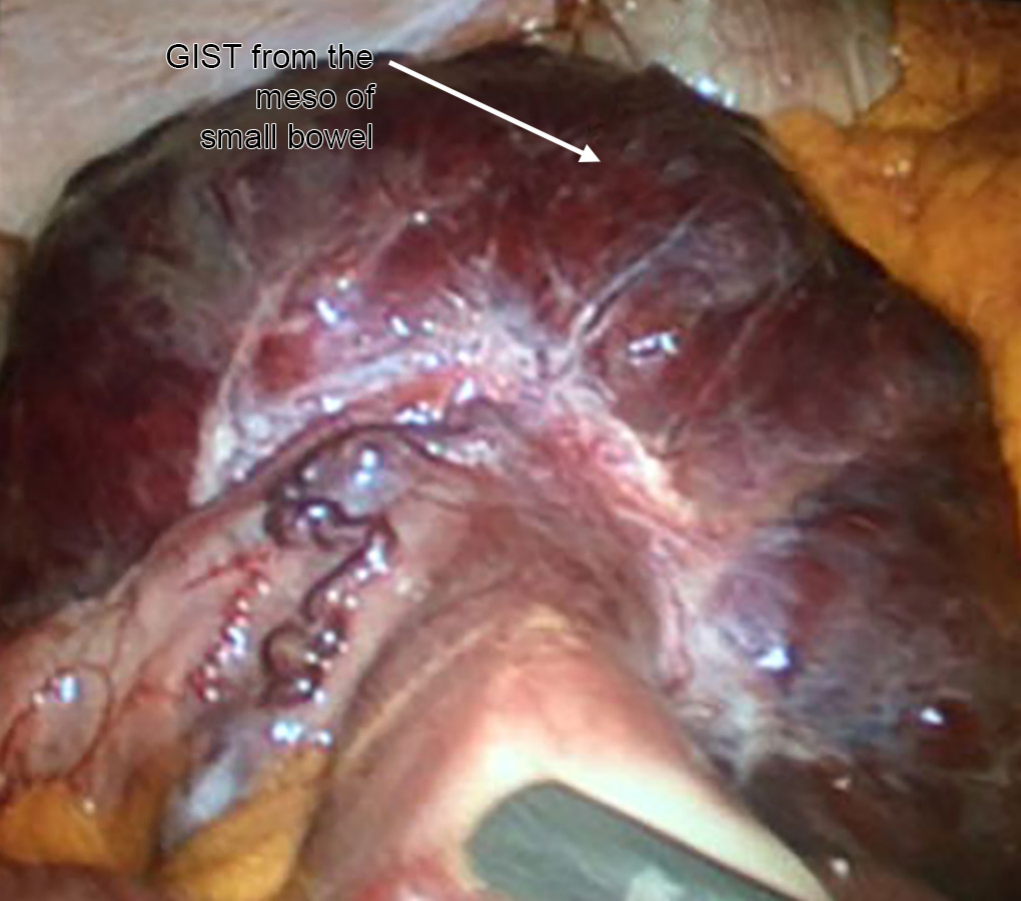
An encapsulated mass lesion originated from the meso of small intestine

A 60-year-old male with a body mass index of 47 kg/m^2^ was admitted to Kecioren Training and Research Hospital, Ankara, Turkey. Type 2 diabetes mellitus and hypertension were present. Laparoscopic sleeve gastrectomy procedure was planned for morbid obesity. Routine laboratory tests, abdominal USG, and upper gastrointestinal endoscopic examination were normal. During the surgery, a red-colored and seemingly encapsulated mass lesion that was thought to be originated from the mesothelium of small bowel, and which was situated under a 4-cm segment of small intestine, was detected (Fig. 2). Exploration was started by open surgical technique, and other sites were found to be clear. The mass and associated intestinal segments were resected, and the remaining open ends were anastomosed. Operation was completed following the drain tube placement. The histopathological examination of the 12.5 × 10 × 7-cm-sized, deep red-colored nodular mass that carried a 4-cm small bowel segment demonstrated that the tumor originated from the intestinal serosal layer. The tumor completely consisted of spindle cells, and the cells showed prominent pleomorphism. Less than five mitoses were observed per 50 high-power microscopic fields. Large areas of necrosis and hemorrhage were present. Immunohistochemically, the tumor cells were positive for CD117, CD99, CD34, and smooth muscle actin (SMA) and negative for S100 and desmin. A diagnosis of GIST was established. Adjuvant chemotherapy with imatinib was administered postoperatively. The patient is still under follow-up by our clinic.

## DISCUSSION

Incidence of cancer is increasing with obesity.^1,6^ In the literature, GIST incidence is reported to be higher among obese patients, who undergo bariatric surgery (0.6–0.8%), than the general population (0.0006–0.0016%).^2^ A majority of GIST cases are older than 50 years. Males and females are equally affected, with a slight male predominance.^7,8^ Our first case was a 52-year-old female, and the second was a 60-year-old male. This suggests that an extensive preoperative abdominal computed tomography (CT) or magnetic resonance imaging (MRI) examination for bariatric surgery candidates older than 50 years old may be useful.

Although these tumors may originate from anywhere along the gastrointestinal tract, the most frequently encountered origin is stomach (60%), followed by small intestine (30%), colon (5%), and rectum (5%).^7-10^ Gastrointestinal stromal tumors with gastric origin are generally localized on fundus or cardia, possibly because of the fact that Cajal’s interstitial cells, the cells of origin of the GISTs, are accumulated in these localizations.^1,2^ Malignity risk categories and prognosis criteria in GISTs are summarized in Table 1.^5^

**Table 1:** **Table 1:** Malignity risk categories and prognosis criteria in GISTs

*Risk category*		*Size*		*Mitotic index*	
Very low risk		<2 cm		<5/50 HPF	
Low risk		2–5 cm		<5/50 HPF	
Moderate risk		<5 cm		6-10/50 HPF	
		5–10 cm		<5/ HPF	
High risk		>10 cm		Any mitotic index	
		Any size		>10/50 HPF	
		>5 cm		>5/ HPF	

HPF: High-power field

In our first case, the GIST originated from gastric fundus, in accordance with the literature. Preoperative endoscopic examination was normal, and the 1.5-cm mass grew exophytically. The initially planned sleeve gastrectomy procedure was performed, as the tumor size was under 2 cm, and the lesion was suitable for being excised within the surgical specimen. Histopathological diagnosis was benign GIST and no further treatment was needed. In our second case, the intraoperatively detected lesion originated from the serosa of small intestine. The mass was larger than 10 cm. The intended bariatric procedure was canceled, since the mass had a high malignancy potential and would possibly require postoperative adjuvant chemotherapy. A complete resection including the tumor was performed. Imatinib chemotherapy was administered postoperatively. Computed tomography controls at 3, 6, and 12 months revealed no signs of metastasis.

Bariatric surgeons should be aware of benign, premalignant, and malignant tumors because of the increased malignancy incidence among obese patients. Preoperative routine workup of obese patients does not include abdominal CT or MRI studies. On the contrary, abdominal USG is usually suboptimal because of extensive fatty tissue; thus, even a 12.5-cm mass growing toward the meso of the small intestine may be overlooked. Routine endoscopic examinations can occasionally detect GISTs with submucosal localization; however, masses with exophytic growth into abdominal cavity may be missed. Therefore, an extensive exploration performed at the beginning of the operation may help in detecting possible tumors.

## References

[1] Chiappetta S, Theodoridou S, Stier C, Weiner RA (2015). Incidental finding of GIST during obesity surgery.. Obes Surg.

[2] Yun HY, Sung R, Kim YC, Choi W, Kim HS, Kim H, Lee GJ, You RY, Park SM, Yun SJ, et al. (2010). Regional distribution of interstitial cells of Cajal(ICC) in human stomach.. Korean J Physiol Pharmacol.

[3] Demetri GD, von Mehren M, Antonescu CR, DeMatteo RP, Ganjoo KN, Maki RG, Pisters PW, Raut CP, Riedel RF, Schuetze S, et al. (2010). NCCN Tasc Force report: update on the management of patients with gastrointestinal stromal tumors.. J Natl Compr Cancer Netw.

[4] Blackstein ME, Blay JY, Corless C, Driman DK, Riddell R, Soulières D, Swallow CJ, Verma S (2006). Canadian advisory committee on GIST gastrointestinal stromal tumors: consensus statement on diagnosis and treatment.. Can J Gastroenterol.

[5] Beltran MA, Pujado B, Méndez PE, Gonzáles FJ, Margulis DI, Contreras MA, Cruces KS (2010). Gastrik gastrointestinal stromal tumor ( GIST) incidentally found and resected during laparoscopic sleeve gastrectomy.. Obes Surg.

[6] Bhaskaran K, Douglas I, Forbes H, dos-Santos-Silva I, Leon DA, Smeeth L (2014). Body-mass index and risk of 22 specific cancers: a population-based cohort study of 5-24 million UK adults.. Lancet.

[7] Yuval JB, Khalaileh A, Abu-Gazala M, Shachar Y, Keidar A, Mintz Y, Nissan A, Elazary R (2014). The true incidence of gastric GIST – a study based on morbidly obese patients undergoing sleeve gastrectomy.. Obes Surg.

[8] DeMatteo RP, Lewis JJ, Leung D, Mudan SS, Woodruff JM, Brennan MF (2000). Two hundred gastrointestinal stromal tumors: recurrence patterns and prognostic factors for survival.. Ann Surg.

[9] Chaudhry UI, DeMatteo RP (2011). Advanced in the surgical management of gastrointestinal stromal tumor.. Adv Surg.

[10] Rabin I, Chikman B, Lavy R, Sandbank J, Maklakovsky M, Gold-Deutch R, Halpren Z, Wassermann I, Halevy A (2009). Gastrointestinal stromal tumors: a 19 year experience.. Isr Med Assoc J.

